# The genome sequence of the Figure of Eighty moth *Tethea ocularis* Linnaeus, 1767

**DOI:** 10.12688/wellcomeopenres.21493.1

**Published:** 2024-06-28

**Authors:** Douglas Boyes, Denise C. Wawman, University of Oxford and Wytham Woods Genome Acquisition Lab, Darwin Tree of Life Barcoding collective, Wellcome Sanger Institute Tree of Life Management, Samples and Laboratory team, Wellcome Sanger Institute Scientific Operations: Sequencing Operations, Wellcome Sanger Institute Tree of Life Core Informatics team, Tree of Life Core Informatics collective, Darwin Tree of Life Consortium

**Affiliations:** 1 UK Centre for Ecology & Hydrology, Wallingford, England, UK; 2 Edward Grey Institute, Department of Biology, University of Oxford, Oxford, England, UK

**Keywords:** Tethea ocularis, Figure of Eighty moth, genome sequence, chromosomal, Lepidoptera

## Abstract

We present a genome assembly from an individual male *Tethea ocularis* (the Figure of Eighty; Arthropoda; Insecta; Lepidoptera; Drepanidae). The genome sequence is 339.1 megabases in span. Most of the assembly is scaffolded into 31 chromosomal pseudomolecules, including the Z sex chromosome. The mitochondrial genome has also been assembled and is 15.28 kilobases in length

## Species taxonomy

Eukaryota; Opisthokonta; Metazoa; Eumetazoa; Bilateria; Protostomia; Ecdysozoa; Panarthropoda; Arthropoda; Mandibulata; Pancrustacea; Hexapoda; Insecta; Dicondylia; Pterygota; Neoptera; Endopterygota; Amphiesmenoptera; Lepidoptera; Glossata; Neolepidoptera; Heteroneura; Ditrysia; Obtectomera; Drepanoidea; Drepanidae; Thyatirinae; *Tethea*; *Tethea ocularis* Linnaeus, 1767(NCBI:txid997289).

## Background

*Tethea ocularis* the Figure of Eighty is a moth in the family Drepanidae, in a group known as lutestrings that was formerly the separate family Thyatiridae. The Drepanidae look similar to the noctuid moths, although they are not as thick-bodied. *Tethea ocularis* has a brown forewing, varying in shade from light to dark brown, fine crosslines and a distinctive white “80” in the centre of the forewing: it is superficially similar to the Poplar Lutestring *Tethea or* and the noctuid Figure of Eight *Diloba caeruleocephala* ( Waring *et al.*, 2017). The darker melanic form of *Tethea ocularis f. fusca* was first recorded in the south and east of England in the 1940’s and has slowly spread across its UK range to become the dominant form ( Skinner, 2009).

The current UK range includes most of south-eastern Scotland, and all of England and Wales, with the exception of Cumberland ( Waring *et al.*, 2017), although it was first recorded in neighbouring Westmoreland in 1983 ( Briggs, 1984). It is not present in Ireland but reached the Isle of Man in 2002 ( Skinner, 2009). *Tethea ocularis* is a species of broad-leaved woodland, hedgerows, parks and gardens, where it is on the wing at night from May to July ( Waring *et al.*, 2017). The larvae feed on aspen *Populus tremula* and other poplars *Populus* spp., in a cocoon between the leaves, from mid-July to September. *Tethea ocularis* overwinters as a pupa ( Waring *et al.*, 2017).

We present a chromosomally complete genome sequence for an adult male *Tethea ocularis*, based on one specimen collected in Wytham Woods, Oxfordshire, as part of the Darwin Tree of Life Project.

## Genome sequence report

**Figure 1.**
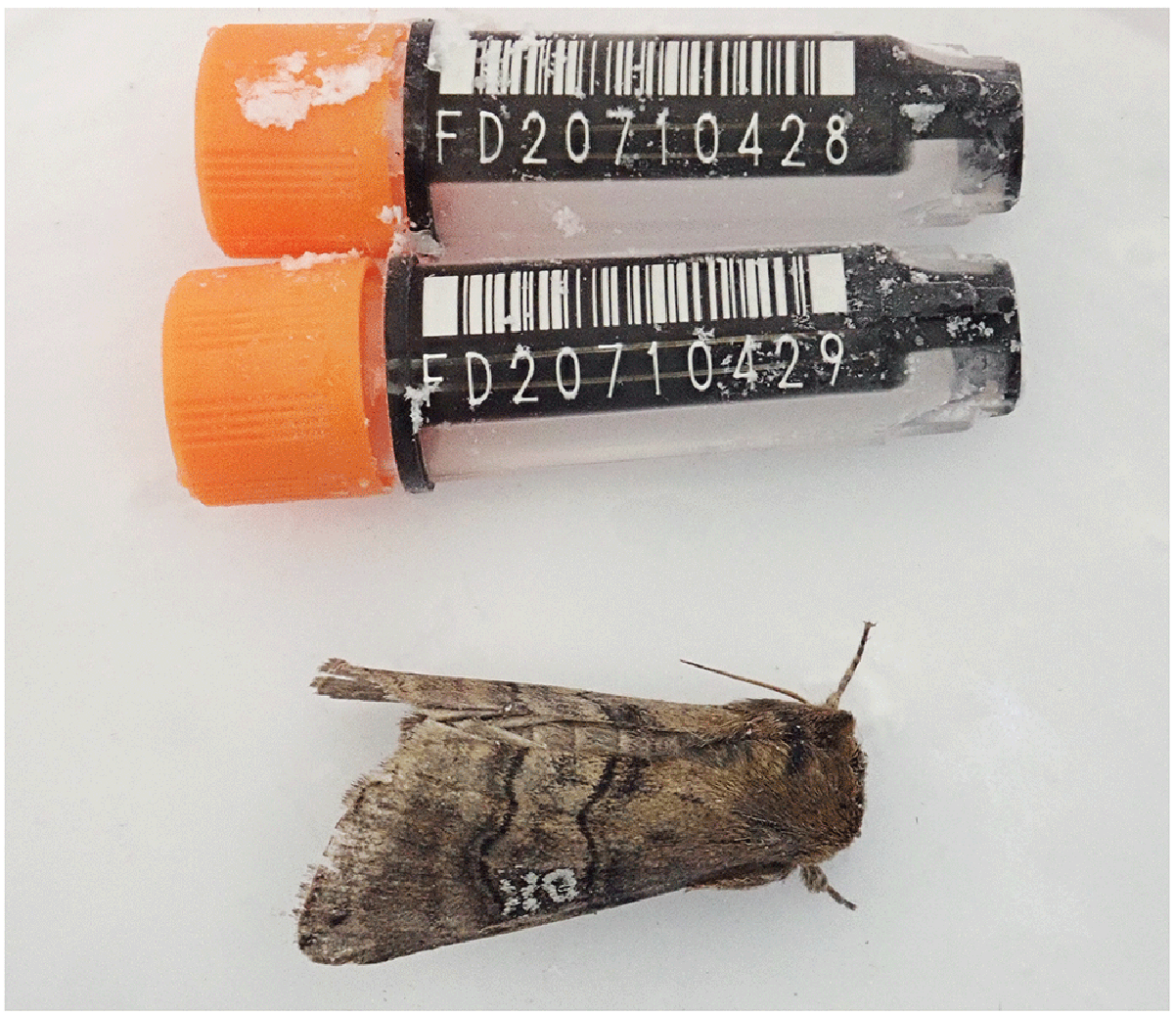
Photograph of the *Tethea ocularis* (ilTetOcul2) specimen used for genome sequencing.

The genome was sequenced from a male *Tethea ocularis* ( Figure 1) collected from Wytham Woods, Oxfordshire, UK (51.77, –1.34). A total of 223-fold coverage in Pacific Biosciences single-molecule HiFi long reads was generated. Primary assembly contigs were scaffolded with chromosome conformation Hi-C data. Manual assembly curation corrected 10 missing joins or mis-joins, increasing the scaffold number by 1.69%.

**Figure 2.**
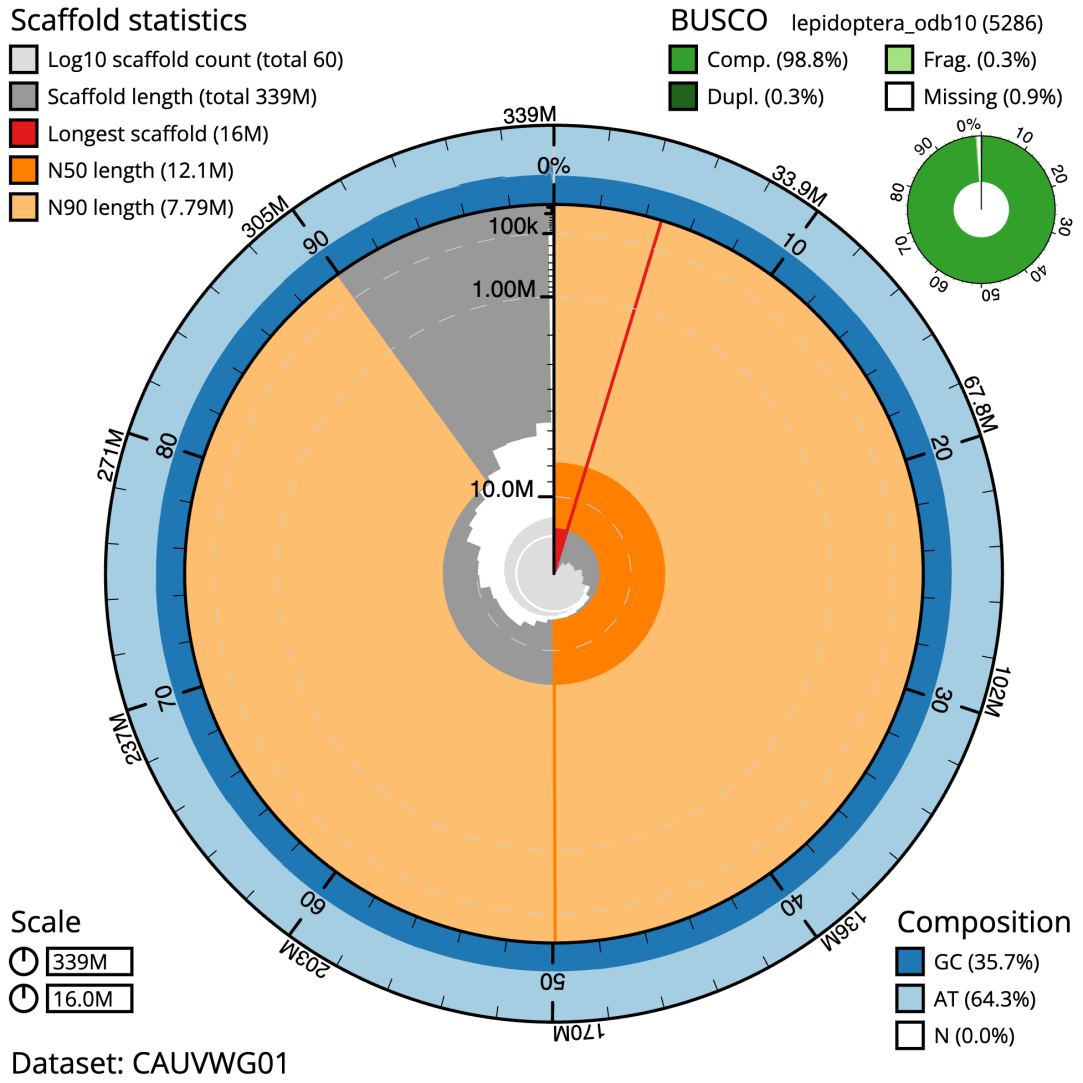
Genome assembly of *Tethea ocularis*, ilTetOcul2.1: metrics. The BlobToolKit snail plot shows N50 metrics and BUSCO gene completeness. The main plot is divided into 1,000 size-ordered bins around the circumference with each bin representing 0.1% of the 339,142,399 bp assembly. The distribution of scaffold lengths is shown in dark grey with the plot radius scaled to the longest scaffold present in the assembly (15,953,683 bp, shown in red). Orange and pale-orange arcs show the N50 and N90 scaffold lengths (12,138,052 and 7,792,192 bp), respectively. The pale grey spiral shows the cumulative scaffold count on a log scale with white scale lines showing successive orders of magnitude. The blue and pale-blue area around the outside of the plot shows the distribution of GC, AT and N percentages in the same bins as the inner plot. A summary of complete, fragmented, duplicated and missing BUSCO genes in the lepidoptera_odb10 set is shown in the top right. An interactive version of this figure is available at https://blobtoolkit.genomehubs.org/view/CAUVWG01/dataset/CAUVWG01/snail.

**Figure 3.**
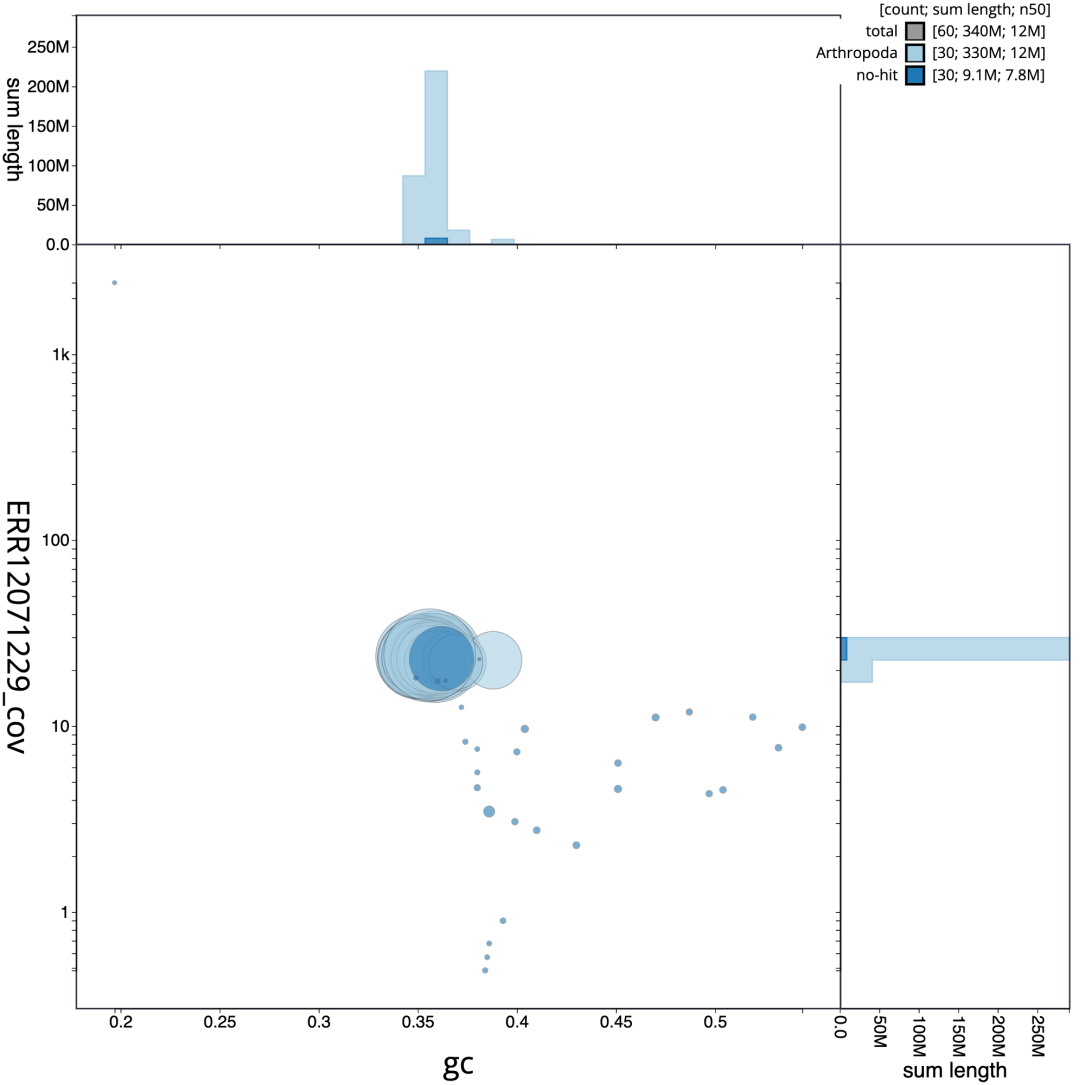
Genome assembly of *Tethea ocularis*, ilTetOcul2.1: BlobToolKit GC-coverage plot. Sequences are coloured by phylum. Circles are sized in proportion to sequence length. Histograms show the distribution of sequence length sum along each axis. An interactive version of this figure is available at https://blobtoolkit.genomehubs.org/view/CAUVWG01/dataset/CAUVWG01/blob.

**Figure 4.**
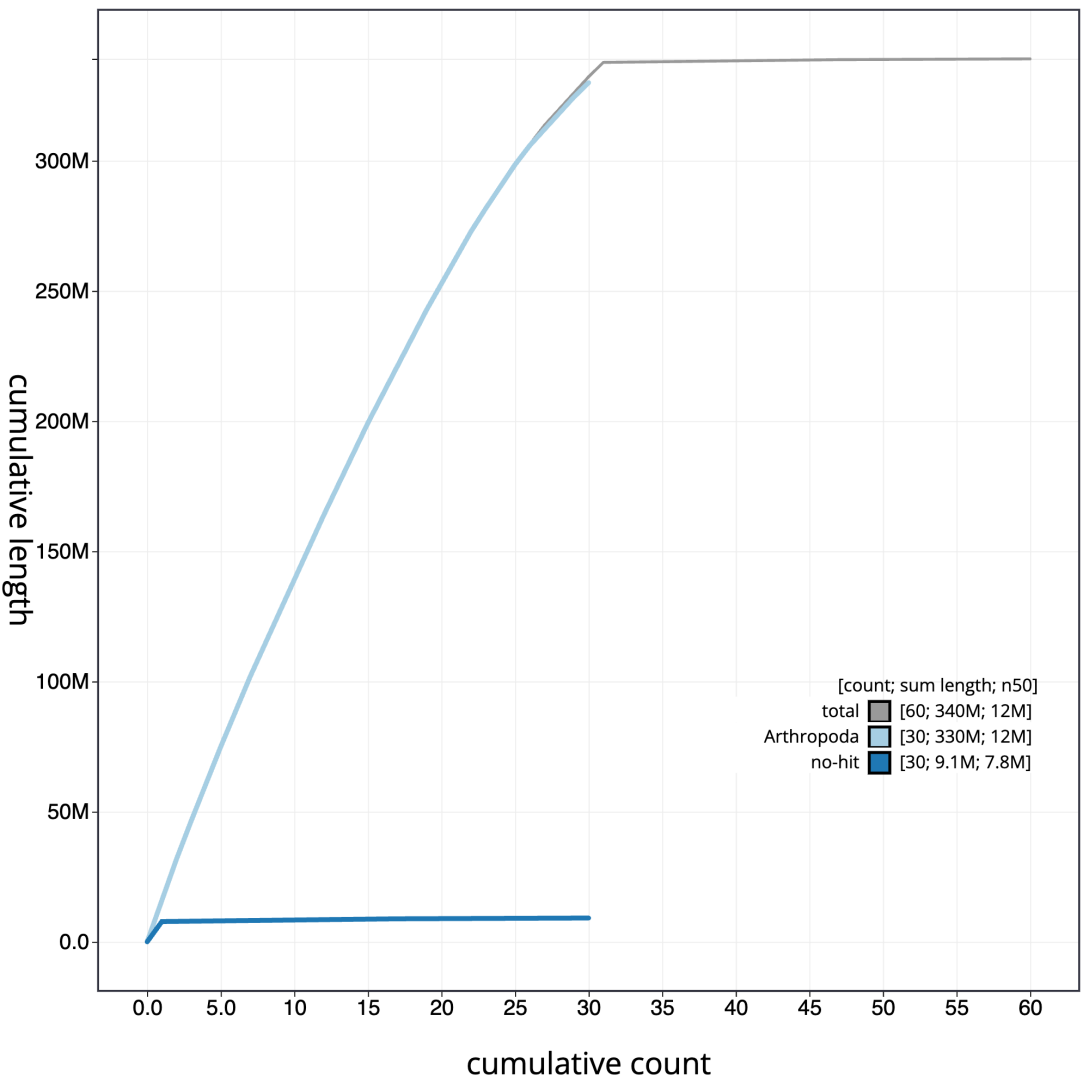
Genome assembly of *Tethea ocularis*, ilTetOcul2.1: BlobToolKit cumulative sequence plot. The grey line shows cumulative length for all sequences. Coloured lines show cumulative lengths of sequences assigned to each phylum using the buscogenes taxrule. An interactive version of this figure is available at https://blobtoolkit.genomehubs.org/view/CAUVWG01/dataset/CAUVWG01/cumulative.

**Figure 5.**
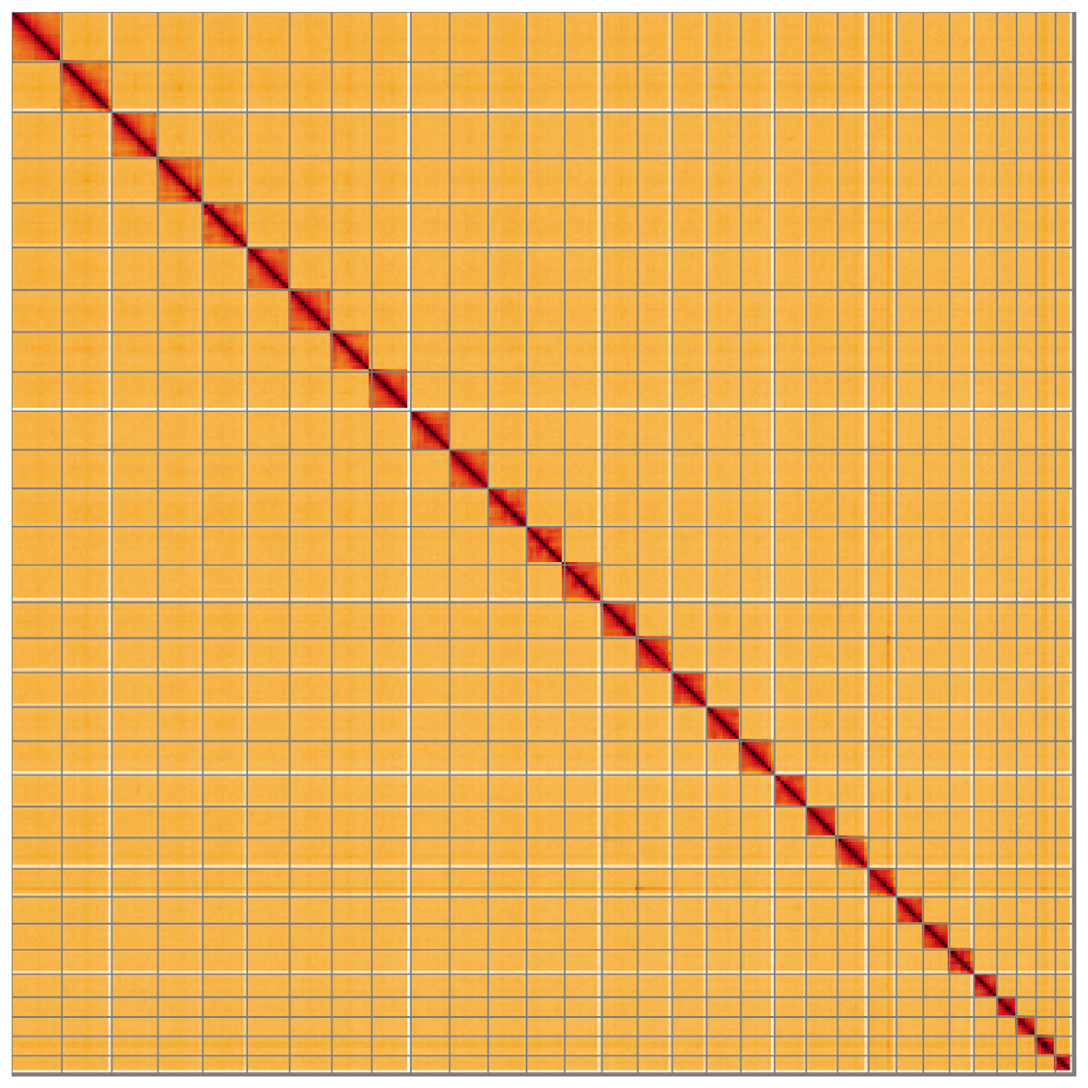
Genome assembly of *Tethea ocularis*, ilTetOcul2.1: Hi-C contact map of the ilTetOcul2.1 assembly, visualised using HiGlass. Chromosomes are shown in order of size from left to right and top to bottom. An interactive version of this figure may be viewed at https://genome-note-higlass.tol.sanger.ac.uk/l/?d=BwYrBgisQ3-PD8AWa8mIQA.

The final assembly has a total length of 339.1 Mb in 59 sequence scaffolds with a scaffold N50 of 12.1 Mb ( Table 1). The snail plot in Figure 2 provides a summary of the assembly statistics, while the distribution of assembly scaffolds on GC proportion and coverage is shown in Figure 3. The cumulative assembly plot in Figure 4 shows curves for subsets of scaffolds assigned to different phyla. Most (99.6%) of the assembly sequence was assigned to 31 chromosomal-level scaffolds, representing 30 autosomes and the Z sex chromosome. Chromosome-scale scaffolds confirmed by the Hi-C data are named in order of size ( Figure 5; Table 2). The Z sex chromosome was assigned by synteny to *Tetheella fluctuosa* (GCA_951216915.1). While not fully phased, the assembly deposited is of one haplotype. Contigs corresponding to the second haplotype have also been deposited. The mitochondrial genome was also assembled and can be found as a contig within the multifasta file of the genome submission.

**Table 1.** Genome data for *Tethea ocularis*, ilTetOcul2.1.

Project accession data		
Assembly identifier	ilTetOcul2.1	
Species	*Tethea ocularis*	
Specimen	ilTetOcul2	
NCBI taxonomy ID	997289	
BioProject	PRJEB66023	
BioSample ID	SAMEA10979182	
Isolate information	ilTetOcul2, male: head and thorax (DNA sequencing) ilTetOcul1: head and thorax (Hi-C sequencing), abdomen (RNA sequencing)	
Assembly metrics *		*Benchmark*
Consensus quality (QV)	63.7	*≥ 50*
*k*-mer completeness	100.0%	*≥ 95%*
BUSCO **	C:98.8%[S:98.4%,D:0.3%], F:0.3%,M:0.9%,n:5,286	*C ≥ 95%*
Percentage of assembly mapped to chromosomes	99.6%	*≥ 95%*
Sex chromosomes	ZZ	*localised homologous pairs*
Organelles	Mitochondrial genome: 15.28 kb	*complete single alleles*
Raw data accessions		
PacificBiosciences Revio	ERR12055563	
Hi-C Illumina	ERR12071233	
PolyA RNA-Seq Illumina	ERR12071234	
Genome assembly		
Assembly accession	GCA_963555595.1	
*Accession of alternate haplotype*	GCA_963555585.1	
Span (Mb)	339.1	
Number of contigs	79	
Contig N50 length (Mb)	10.0	
Number of scaffolds	59	
Scaffold N50 length (Mb)	12.1	
Longest scaffold (Mb)	15.95	

* Assembly metric benchmarks are adapted from column VGP-2020 of “Table 1: Proposed standards and metrics for defining genome assembly quality” from Rhie *et al.* (2021). ** BUSCO scores based on the lepidoptera_odb10 BUSCO set using version 5.3.2. C = complete [S = single copy, D = duplicated], F = fragmented, M = missing, n = number of orthologues in comparison. A full set of BUSCO scores is available at https://blobtoolkit.genomehubs.org/view/CAUVWG01/dataset/CAUVWG01/busco.

**Table 2.** Chromosomal pseudomolecules in the genome assembly of *Tethea ocularis*, ilTetOcul2.

INSDC accession	Chromosome	Length (Mb)	GC%
OY741957.1	1	15.91	36.0
OY741958.1	2	14.69	35.5
OY741959.1	3	14.26	36.0
OY741960.1	4	14.13	35.5
OY741961.1	5	13.54	35.0
OY741962.1	6	13.4	35.0
OY741963.1	7	12.75	35.5
OY741964.1	8	12.41	35.5
OY741965.1	9	12.37	35.0
OY741966.1	10	12.27	35.5
OY741967.1	11	12.22	35.5
OY741968.1	12	12.14	35.0
OY741969.1	13	11.87	35.0
OY741970.1	14	11.4	35.5
OY741971.1	15	10.96	35.5
OY741972.1	16	10.93	35.0
OY741973.1	17	10.91	36.0
OY741974.1	18	10.75	35.5
OY741975.1	19	10.08	36.0
OY741976.1	20	9.99	35.5
OY741977.1	21	9.86	36.0
OY741978.1	22	8.86	36.0
OY741979.1	23	8.55	36.0
OY741980.1	24	8.34	35.5
OY741981.1	25	7.79	36.0
OY741982.1	26	7.35	36.0
OY741983.1	27	6.25	36.5
OY741984.1	28	6.19	37.0
OY741985.1	29	6.17	39.0
OY741986.1	30	5.51	37.0
OY741956.1	Z	15.95	35.5
OY741987.1	MT	0.02	19.5

The estimated Quality Value (QV) of the final assembly is 63.7 with *k*-mer completeness of 100.0%, and the assembly has a BUSCO v5.3.2 completeness of 98.8% (single = 98.4%, duplicated = 0.3%), using the lepidoptera_odb10 reference set ( *n* = 5,286).

Metadata for specimens, barcode results, spectra estimates, sequencing runs, contaminants and pre-curation assembly statistics are given at https://links.tol.sanger.ac.uk/species/997289.

## Methods

### Sample acquisition and nucleic acid extraction

A male *Tethea ocularis* (specimen ID Ox001919, ToLID ilTetOcul2) was collected from Wytham Woods, Oxfordshire (biological vice-county Berkshire), UK (latitude 51.77, longitude –1.34) on 2021-06-16. The specimen used for Hi-C and RNA sequencing (specimen ID Ox000465, ToLID ilTetOcul1) was collected from the same location on 2020-06-13. Both specimens were collected and identified by Douglas Boyes (University of Oxford) and preserved on dry ice.

The workflow for high molecular weight (HMW) DNA extraction at the Wellcome Sanger Institute (WSI) includes a sequence of core procedures: sample preparation; sample homogenisation, DNA extraction, fragmentation, and clean-up. The sample was prepared for extraction in the WSI Tree of Life Core Laboratory: the ilTetOcul2 sample was weighed and dissected on dry ice ( Jay *et al.*, 2023), and tissue from the head and thorax was homogenised using a PowerMasher II tissue disruptor ( Denton *et al.*, 2023a).

HMW DNA was extracted in the WSI Scientific Operations core using the Automated MagAttract v2 protocol ( Oatley *et al.*, 2023a). The DNA was sheared into an average fragment size of 12–20 kb in a Megaruptor 3 system with speed setting 31 ( Bates *et al.*, 2023). Sheared DNA was purified by solid-phase reversible immobilisation ( Strickland *et al.*, 2023a): in brief, the method employs a 1.8X ratio of AMPure PB beads to sample to eliminate shorter fragments and concentrate the DNA. The concentration of the sheared and purified DNA was assessed using a Nanodrop spectrophotometer and Qubit Fluorometer and Qubit dsDNA High Sensitivity Assay kit. Fragment size distribution was evaluated by running the sample on the FemtoPulse system.

RNA was extracted from abdomen tissue of ilTetOcul1 in the Tree of Life Laboratory at the WSI using the RNA Extraction: Automated MagMax™ *mir*Vana protocol ( do Amaral *et al.*, 2023). The RNA concentration was assessed using a Nanodrop spectrophotometer and a Qubit Fluorometer using the Qubit RNA Broad-Range Assay kit. Analysis of the integrity of the RNA was done using the Agilent RNA 6000 Pico Kit and Eukaryotic Total RNA assay.

Protocols developed by the WSI Tree of Life laboratory are publicly available on protocols.io ( Denton *et al.*, 2023b).

### Sequencing

Pacific Biosciences HiFi circular consensus DNA sequencing libraries were constructed according to the manufacturers’ instructions. Poly(A) RNA-Seq libraries were constructed using the NEB Ultra II RNA Library Prep kit. DNA and RNA sequencing was performed by the Scientific Operations core at the WSI on Pacific Biosciences Revio (HiFi) and Illumina HiSeq 4000 (RNA-Seq) instruments. Hi-C data were also generated from head and thorax tissue of ilTetOcul1 using the Arima2 kit and sequenced on the HiSeq X Ten instrument.

### Genome assembly, curation and evaluation

Assembly was carried out with Hifiasm ( Cheng *et al.*, 2021) and haplotypic duplication was identified and removed with purge_dups ( Guan *et al.*, 2020). The assembly was then scaffolded with Hi-C data ( Rao *et al.*, 2014) using YaHS ( Zhou *et al.*, 2023). The assembly was checked for contamination and corrected using the gEVAL system ( Chow *et al.*, 2016) as described previously ( Howe *et al.*, 2021). Manual curation was performed using gEVAL, HiGlass ( Kerpedjiev *et al.*, 2018) and PretextView ( Harry, 2022). The mitochondrial genome was assembled using MitoHiFi ( Uliano-Silva *et al.*, 2023), which runs MitoFinder ( Allio *et al.*, 2020) or MITOS ( Bernt *et al.*, 2013) and uses these annotations to select the final mitochondrial contig and to ensure the general quality of the sequence.

A Hi-C map for the final assembly was produced using bwa-mem2 ( Vasimuddin *et al.*, 2019) in the Cooler file format ( Abdennur & Mirny, 2020). To assess the assembly metrics, the *k*-mer completeness and QV consensus quality values were calculated in Merqury ( Rhie *et al.*, 2020). This work was done using Nextflow ( Di Tommaso *et al.*, 2017) DSL2 pipelines “sanger-tol/readmapping” ( Surana *et al.*, 2023a) and “sanger-tol/genomenote” ( Surana *et al.*, 2023b). The genome was analysed within the BlobToolKit environment ( Challis *et al.*, 2020) and BUSCO scores ( Manni *et al.*, 2021; Simão *et al.*, 2015) were calculated.

Table 3 contains a list of relevant software tool versions and sources.

**Table 3.** Software tools: versions and sources.

Software tool	Version	Source
BlobToolKit	4.2.1	https://github.com/blobtoolkit/blobtoolkit
BUSCO	5.3.2	https://gitlab.com/ezlab/busco
Hifiasm	0.19.5-r587	https://github.com/chhylp123/hifiasm
HiGlass	1.11.6	https://github.com/higlass/higlass
Merqury	MerquryFK	https://github.com/thegenemyers/MERQURY.FK
MitoHiFi	3	https://github.com/marcelauliano/MitoHiFi
PretextView	0.2	https://github.com/wtsi-hpag/PretextView
purge_dups	1.2.5	https://github.com/dfguan/purge_dups
sanger-tol/genomenote	v1.0	https://github.com/sanger-tol/genomenote
sanger-tol/readmapping	1.1.0	https://github.com/sanger-tol/readmapping/tree/1.1.0
YaHS	1.2a.2	https://github.com/c-zhou/yahs

### Wellcome Sanger Institute – Legal and Governance

The materials that have contributed to this genome note have been supplied by a Darwin Tree of Life Partner. The submission of materials by a Darwin Tree of Life Partner is subject to the **‘Darwin Tree of Life Project Sampling Code of Practice’**, which can be found in full on the Darwin Tree of Life website here. By agreeing with and signing up to the Sampling Code of Practice, the Darwin Tree of Life Partner agrees they will meet the legal and ethical requirements and standards set out within this document in respect of all samples acquired for, and supplied to, the Darwin Tree of Life Project. 

Further, the Wellcome Sanger Institute employs a process whereby due diligence is carried out proportionate to the nature of the materials themselves, and the circumstances under which they have been/are to be collected and provided for use. The purpose of this is to address and mitigate any potential legal and/or ethical implications of receipt and use of the materials as part of the research project, and to ensure that in doing so we align with best practice wherever possible. The overarching areas of consideration are:

•      Ethical review of provenance and sourcing of the material

•      Legality of collection, transfer and use (national and international)

Each transfer of samples is further undertaken according to a Research Collaboration Agreement or Material Transfer Agreement entered into by the Darwin Tree of Life Partner, Genome Research Limited (operating as the Wellcome Sanger Institute), and in some circumstances other Darwin Tree of Life collaborators.

## Data Availability

European Nucleotide Archive: *Tethea ocularis* (figure of eighty). Accession number PRJEB66023; https://identifiers.org/ena.embl/PRJEB66023 ( Wellcome Sanger Institute, 2023). The genome sequence is released openly for reuse. The *Tethea ocularis* genome sequencing initiative is part of the Darwin Tree of Life (DToL) project. All raw sequence data and the assembly have been deposited in INSDC databases. The genome will be annotated using available RNA-Seq data and presented through the Ensembl pipeline at the European Bioinformatics Institute. Raw data and assembly accession identifiers are reported in Table 1.

## Author information

Members of the University of Oxford and Wytham Woods Genome Acquisition Lab are listed here: https://doi.org/10.5281/zenodo.7125292.

Members of the Darwin Tree of Life Barcoding collective are listed here: https://doi.org/10.5281/zenodo.4893703.

Members of the Wellcome Sanger Institute Tree of Life Management, Samples and Laboratory team are listed here: https://doi.org/10.5281/zenodo.10066175.

Members of Wellcome Sanger Institute Scientific Operations: Sequencing Operations are listed here: https://doi.org/10.5281/zenodo.10043364.

Members of the Wellcome Sanger Institute Tree of Life Core Informatics team are listed here: https://doi.org/10.5281/zenodo.10066637.

Members of the Tree of Life Core Informatics collective are listed here: https://doi.org/10.5281/zenodo.5013541.

Members of the Darwin Tree of Life Consortium are listed here: https://doi.org/10.5281/zenodo.4783558.
