# Supplementary material for: The genome sequence of the Figure of Eighty moth Tethea ocularis Linnaeus, 1767
Source: Wellcome Open Res. 2024 Jun 28;9:348. [Version 1] doi: 10.12688/wellcomeopenres.21493.1 (PMC11416703; doi:10.12688/wellcomeopenres.21493.1)
Supplement: Peer review history

# Reviewer report: Reviewer response for version 1

**DOI:** 10.21956/wellcomeopenres.23763.r93819

**Published:** 2024-09-21

**Authors:** Panagiotis Ioannidis

**Affiliations:** 1 Foundation for Research & Technology - Hellas, Heraklion, Crete, GR 70013, Greece

The herein reported genome sequence of the *Tethea ocularis *moth is a high-quality assembly which is based on deep, PacBio sequencing (223-fold coverage). Additionally, BUSCO scores are also very good at almost 99%.

 I expected that this genome is ready to be used for downstream analyses and as such I fully endorse this manuscript in its current form.

Are sufficient details of methods and materials provided to allow replication by others?

Yes

Is the rationale for creating the dataset(s) clearly described?

Yes

Are the datasets clearly presented in a useable and accessible format?

Yes

Are the protocols appropriate and is the work technically sound?

Yes

Reviewer Expertise:

Insect genomics, bioinformatics

I confirm that I have read this submission and believe that I have an appropriate level of expertise to confirm that it is of an acceptable scientific standard.

# Reviewer report: Reviewer response for version 1

**DOI:** 10.21956/wellcomeopenres.23763.r94816

**Published:** 2024-09-18

**Authors:** Antonio S. Ortiz

**Affiliations:** 1 University of Murcia, Murcia, Spain

Boyes & Wawman describes the genome assembly of *Tethea ocularis *and provides important genomic information about its size (near 340 Mb) in 31 chromosomes.

 A new genome sequenced from the moth family Drepanidae is added to others as

- *Tethea albicostata* (Bremer 1861) by Liang *et al*. (2023) published in Mitochondrial DNA part B,
- *Drepana arcuata* Walker, 1855 by Yadav *et al*. (2020) published in Data in Brief and
- *Drepana falcataria* (Linnaeus, 1758) by Boyes & Lewis (2023),
- *Watsonalla binaria *(Hufnagel, 1767) by Boyes & Chua (2023) and
- *Achlya flavicornis* (Linnaeus, 1758) by Crowley & Phillips (2023), the last three published in Wellcome Open Research (WOR).
  This assembly provides valuable infomation for the genomics of Lepidoptera and for further phylognetic research in the evolutionary pattern in Lepidoptera.

The methods for the sequencing and assembly used are appropriate and technically supported but It would be interesting to offer a calculation of the number of genes that encode proteins.

 I suggest the addition of author names and year in the first mention of the species in the text ( *Tethea ocularis, Tethea or, Diloba caeruleocephala*, *T. ocularis* f. *fusca* and *Tetheella fluctuosa*).

Are sufficient details of methods and materials provided to allow replication by others?

Yes

Is the rationale for creating the dataset(s) clearly described?

Yes

Are the datasets clearly presented in a useable and accessible format?

Yes

Are the protocols appropriate and is the work technically sound?

Yes

Reviewer Expertise:

Molecular genetics on Lepidoptera with special interest in evolutionary proccesses.

I confirm that I have read this submission and believe that I have an appropriate level of expertise to confirm that it is of an acceptable scientific standard.

## References

[1] : An updated checklist of Thyatirinae (Lepidoptera, Drepanidae) from China, with descriptions of one new species. *Zootaxa* .2015;3941(1) : 10.11646/zootaxa.3941.1.1 1-48 10.11646/zootaxa.3941.1.1 25947492

[2] : Transcriptome analysis of a social caterpillar, Drepana arcuata: De novo assembly, functional annotation and developmental analysis. *PLoS One* .2020;15(6) : 10.1371/journal.pone.0234903 e0234903 10.1371/journal.pone.0234903 32569288 PMC7307738

[3] : The genome sequence of the Pebble Hook-tip, Drepana falcataria (Linnaeus, 1758). *Wellcome Open Res* .2023;8: 10.12688/wellcomeopenres.19213.1 207 10.12688/wellcomeopenres.19213.1 38764970 PMC11101918

[4] : The genome sequence of the Oak Hook-tip, Watsonalla binaria (Hufnagel, 1767). *Wellcome Open Res* .2023;8: 10.12688/wellcomeopenres.19634.1 324 10.12688/wellcomeopenres.19634.1 37885811 PMC10598455

[5] : The genome sequence of the Yellow Horned, Achlya flavicornis (Linnaeus, 1758). *Wellcome Open Research* .2023;8: 10.12688/wellcomeopenres.19846.1 10.12688/wellcomeopenres.19846.1 PMC1185100340008385
